# Procalcitonin as a prognostic biomarker of severe sepsis and septic shock

**DOI:** 10.1186/cc12668

**Published:** 2013-06-19

**Authors:** JRA de Azevedo, OJM Torres, O Malafaia

**Affiliations:** 1 Hospital São Domingos, Bequimão, São Luis, MA, Brazil

## Introduction

Procalcitonin (PCT), the precursor peptide of calcitonin, has extremely low levels in healthy subjects. In response to bacterial infectious stimulation, PCT serum levels rise substantially. Recently PCT has been used as a biomarker for prognosis of severe sepsis and septic shock. Some studies have shown that isolated high levels do not predict outcome. Encouraging results were obtained with the evaluation of serum PCT levels. The objective of this study was to evaluate the tendency of the plasma concentration and clearance of PCT as biomarkers for prognosis of patients with severe sepsis and septic shock, compared with another early prognosis marker, the number of SIRS criteria at sepsis diagnosis.

## Methods

We conducted a prospective, observational, cohort study, with patients with severe sepsis and septic shock. The serum procalcitonin was determined at diagnosis of sepsis and after 24 and 48 hours. Demographic data, APACHE IV score, SOFA score on arrival, number of SIRS criteria at diagnosis, site of infection and microbiological results were recorded.

## Results

Twenty-eight patients were included, 19 clinical and nine surgical. In 13 patients (46.4%) the source of sepsis was pulmonary, abdominal in seven cases (25.0%), urinary in five cases (17.9%) and soft tissue in three cases (10.7%). Fifteen patients had severe sepsis and 13 septic shock. Overall mortality was 17.9% (five patients), three with septic shock. Twenty-eight PCT determinations were performed at sepsis diagnosis, 27 after 24 hours and 26 after 48 hours. The initial concentration was not significantly different between the survivor and nonsurvivor groups, but the differences between the two groups after 24 and 48 hours were statistically significant. There was no difference in the number of SIRS criteria. The 24-hour procalcitonin clearance proved to be significantly higher in the group of survivors (-3.0 vs. -300.0, *P *= 0.028). Although the 48-hour procalcitonin clearance was shown to be higher in the group of survivors when compared with nonsurvivors, the difference did not reach statistical significance.

## Conclusion

Persistently high PCT concentrations in plasma, as well as reduced 24-hour PCT clearance, were associated with a significant increase in mortality in patients with severe sepsis and septic shock.

